# Circulating Levels of Betatrophin and Irisin Are Not Associated with Pancreatic *β*-Cell Function in Previously Diagnosed Type 2 Diabetes Mellitus Patients

**DOI:** 10.1155/2016/2616539

**Published:** 2015-11-16

**Authors:** Lingshu Wang, Jun Song, Chuan Wang, Peng Lin, Kai Liang, Yu Sun, Tianyi He, Wenjuan Li, Ruxing Zhao, Jun Qin, Yiran Lu, Jinbo Liu, Fuqiang Liu, Xinguo Hou, Li Chen

**Affiliations:** Department of Endocrinology, Qilu Hospital of Shandong University, Jinan, Shandong 250012, China

## Abstract

Betatrophin and irisin are two recently identified hormones which may participate in regulating pancreatic *β*-cell function. However, the associations of these two hormones with *β*-cell function remain unclear. The present study aims to demonstrate the associations of circulating betatrophin and irisin levels with *β*-cell function, assessed by the area under the curve (AUC) of C-peptide, and the possible correlation between these two hormones in previously diagnosed type 2 diabetes mellitus (T2DM) patients. In total, 20 age-, sex-, and body mass index- (BMI-) matched normal glucose tolerance (NGT) subjects and 120 previously diagnosed T2DM patients were included in this study. Partial correlation analysis was used to evaluate the relationships between these two hormones and indexes of *β*-cell function and insulin resistance. Our results showed that betatrophin levels were significantly elevated, while irisin levels were significantly decreased, in patients with T2DM compared with NGT subjects. However, partial correlation analysis showed that betatrophin levels did not correlate with *β*-cell function-related variables or insulin resistance-related variables before or after controlling multiple covariates, while irisin correlated positively with insulin sensitivity but is not associated with *β*-cell function-related variables. Besides, no correlation was observed between betatrophin and irisin levels. Hence we concluded that betatrophin and irisin were not associated with *β*-cell function in previously diagnosed T2DM patients.

## 1. Introduction

It has been suggested that the best treatment, and a potential cure, for both type 1 mellitus (T1DM) and type 2 diabetes mellitus (T2DM), is to replace or regenerate the pancreatic *β*-cell mass [1]. Interestingly, two recently identified hormones, betatrophin and irisin, might be involved in this process [2], although their specific physiological effects on pancreatic *β*-cell have not been confirmed. Betatrophin (also known as ANGPTL8, Lipasin, RIFL, EG624219, and TD26) [3, 4] was found to be a novel stimulator of *β*-cell by Yi et al. in a S961-induced insulin resistance mouse model [5]. Overexpression of betatrophin in mice livers was reported to induce a striking increase of *β*-cell proliferation rate 17-fold higher than the controls. However these inspiring discoveries were later challenged by either genetic ablation of betatrophin or its overexpression, which showed no significant effect on *β*-cell mass in mice [6, 7]. Despite of the facts above, studies of betatrophin in human subjects were also limited and nonconsistent, and researches focusing on correlation between betatrophin and *β*-cell function in T2DM human subjects were lacking. Therefore, the relevance of betatrophin with *β*-cell function needs to be further warranted, especially in human subjects.

Irisin was initially described as a protective factor against diet-induced weight gain by browning of white adipose tissue (WAT) [8]. Interestingly, a moderate increase in circulating irisin levels could also improve diet-induced insulin resistance [8], which indicated the potential important role of irisin in regulating glucose homeostasis. However, only few studies explore the association of irisin with *β*-cell function, although there have been numerous studies conducted in diabetes subjects. A recent study reported that serum irisin levels were closely related to homeostasis model assessment-*β* (HOMA-*β*) in normal glucose tolerance (NGT) subjects, suggesting that irisin may play a crucial role in *β*-cell function. However, similar relationship was not observed in diabetic subjects [9]. Considering that HOMA-*β* could not reflect *β*-cell function accurately, the relationship between irisin and *β*-cell function, assessed by a more accurate method, in diabetes subjects remains unclear and needs further investigation.

Furthermore, Zhang et al. discovered that irisin could promote the expression of betatrophin, thus raising the possibility that the euglycemic effect of irisin was partially mediated by the upregulation of betatrophin through *β*-cell proliferation [10]. However, the relationship between betatrophin and irisin in human subjects has not been clarified. Therefore, the present study aims to demonstrate the association of circulating betatrophin and irisin levels with pancreatic *β*-cell function, assessed by the gold standard measure of *β*-cell function, the area under the curve (AUC) of C-peptide [11], and the possible correlation between betatrophin and irisin levels in previously diagnosed T2DM patients.

## 2. Methods

### 2.1. Subjects

This cross-sectional study randomly recruited 120 previously diagnosed T2DM patients with durations ≥ 1 year at Qilu Hospital of Shandong University from May 2014 to November 2014 after a review of their medical records based on the following 1999 WHO criteria: fasting blood glucose (FBG) ≥ 126 mg/dL (7.0 mmol/L) and/or 2 h postprandial blood glucose ≥ 200 mg/dL (11.1 mmol/L) [12]. The following exclusion criteria were applied: patients with (1) T1DM, secondary diabetes, or specific types of diabetes or diabetic ketoacidosis, lactic acidosis, or a hyperglycemic hyperosmolar state; (2) Diabetic foot or inflammatory or infectious diseases; (3) acute cerebral infarction or acute myocardial infarction; (4) familial hypercholesterolemia, and samples with visible lipidemia and hemolysis; and (5) heart failure and severely impaired liver or renal function. Healthy age-, sex-, and body mass index- (BMI-) matched normal glucose tolerance (NGT) controls (*n* = 20), without a family history of T2DM, were recruited by advertising. Written informed consent was obtained from all subjects, and the study was approved by the ethics committee of the Qilu Hospital of Shandong University.

### 2.2. Clinical Data Collection

The computerized patient record system of Qilu Hospital was used to collect data regarding the demographic characteristics and previous medical histories of subjects. Antidiabetic medications were included in the following categories: insulin, insulin secretagogues, and others (thiazolidinedione (TZD), metformin, and alpha glucosidase inhibitor). BMI was determined by dividing the weight by the height squared (kg/m^2^). Blood pressure (BP) was measured 3 consecutive times (OMRON Model HEM-752 FUZZY, Omron Company, Dalian, China) using the left arm after the subject had remained seated for at least 5 min, and the average reading was used for the analysis. Fasting blood samples were collected after a 10-hour fast and before the ingestion of breakfast and medication. FBG, total cholesterol (TC), triglycerides (TGs), low-density lipoprotein cholesterol (LDL-C), and high-density lipoprotein cholesterol (HDL-C) levels were measured by an automatic analyzer (TOSHIBA TBA-40F, Toshiba, Japan). HbA1C was measured by high-performance liquid chromatography (BIO–RAD VARIANT II, Bio-Rad, USA). Plasma betatrophin levels were determined by an ELISA (Wuhan Eiaab Science, Wuhan, China; Catalogue number E11644h). Plasma irisin levels were measured by ELISA (Phoenix Pharmaceuticals, Inc., Burlingame, USA; Catalogue number EK-067-29).

### 2.3. Islet *β*-Cell Function and Insulin Resistance Assessment

Any insulin treatment was stopped 10 hours before collecting fasting blood samples. 75-g oral glucose tolerance test (OGTT) was carried out for all T2DM patients and blood glucose and C-peptide were measured at 0, 30, 60, and 120 min after glucose administration. Plasma C-peptide levels were detected by a chemiluminescence immunoassay analyzer (Bayer ADVIA Centaur, Bayer, Germany). *β*-cell function was presented by AUC (including AUC_0–0.5_, AUC_0-1_, and AUC_0–2_) of C-peptide release test calculated using the trapezoidal rule. Fasting C-peptide and glucose was used to calculate homoeostasis model assessment to estimate basal *β*-cell function (HOMA2-%B), insulin sensitivity (HOMA2-%S), and insulin resistance (HOMA2-IR) by the computerized HOMA2 model downloaded from http://www.ocdem.ox.ac.uk/ [13].

### 2.4. Statistical Analysis

The continuous variables with normal distribution are expressed as the mean ± standard error of mean (SEM), and the variables with nonnormal distribution are presented as the median (interquartile range). The categorical variables are presented as numbers (%). Normal distribution of the data was tested using the Kolmogorov-Smirnov test. Between-group differences were detected using one-way ANOVA (LSD) test (the continuous variables in normal distribution), Mann Whitney *U*-test (the skewed continuous variables), or chi-squared test (categorical variables). The correlations between variables were assessed using a Pearson correlation analysis by controlling for the covariates. *P* < 0.05 was considered statistically significant. All the above statistical analyses were performed with SPSS 16.0 software (SPSS Inc., Chicago, USA).

## 3. Results

### 3.1. Comparison of Betatrophin and Irisin Levels in NGT and T2DM Subjects

The glucose-related variables and levels of betatrophin and irisin in NGT and T2DM subjects were shown in Table 1. As expected, T2DM subjects showed higher FBG and HbA1c levels, compared with age-, sex- and BMI-matched NGT group. However, the levels of fasting insulin of T2DM subjects were also higher, although the fasting C-peptide levels were not significantly different between two groups. Notably, circulating betatrophin levels were significantly elevated (592.1 ± 37.5 versus 291.1 ± 37.3 pg/mL), while irisin levels were significantly decreased (3.4 ± 0.1 versus 4.7 ± 0.1 ng/mL), in patients with T2DM compared with NGT subjects.

### 3.2. Comparison of Betatrophin and Irisin Levels in T2DM Subjects with Different *β*-Cell Function

The clinical characteristics and levels of betatrophin and irisin in T2DM subjects with different *β*-cell function were shown in Table 2. *β*-cell function was measured by the gold standard measure, the AUC of C-peptide [11], and we grouped the subjects to four quartiles according to the AUC of C-peptide levels [14]. In general, levels of BMI, fasting C-peptide, HOMA2-%B, HOMA2-IR, and TG were found to be positively associated with AUC_0–2_ values, and HbA1c, HOMA2-%S, the duration of diabetes, the percentage of insulin usage, and HDL-C levels were inversely associated. No significant differences among the groups in their BP, FBG, TC, and LDL-C were found. Notably, circulating levels of betatrophin and irisin were not significantly different among these four groups.

### 3.3. Correlations between Betatrophin Levels and Indexes of *β*-Cell Function and Insulin Resistance

**Figure 1:**
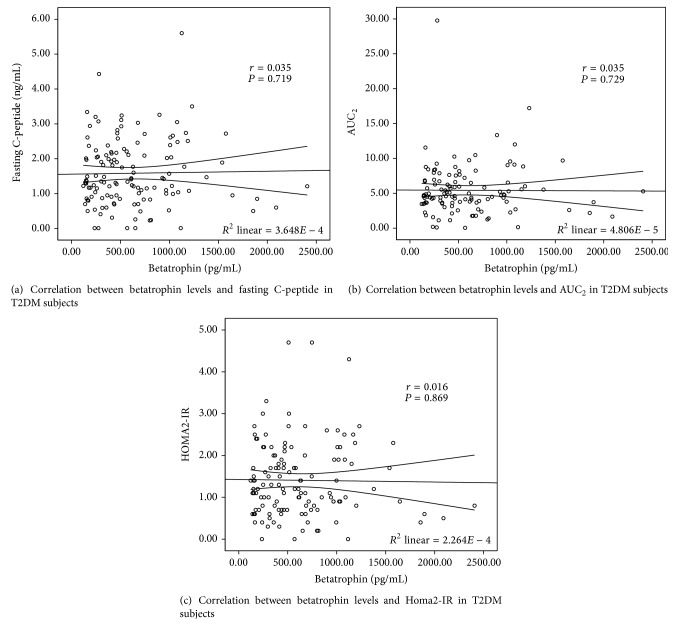
Correlation between betatrophin levels and glucose-related variables in T2DM subjects.

The correlations between betatrophin levels and glucose-related variables based on the values of 120 individual T2DM subjects were shown in Table 3 and Figure 1. We observed that betatrophin levels did not correlate with *β*-cell function-related variables (AUC_0–0.5_, AUC_0-1_, AUC_0–2_, and HOMA2-%B) or insulin resistance-related variables (HOMA2-%S and HOMA2-IR) in T2DM subjects before or after controlling multiple covariates.

### 3.4. Correlations between Irisin Levels and Indexes of *β*-Cell Function and Insulin Resistance

**Figure 2:**
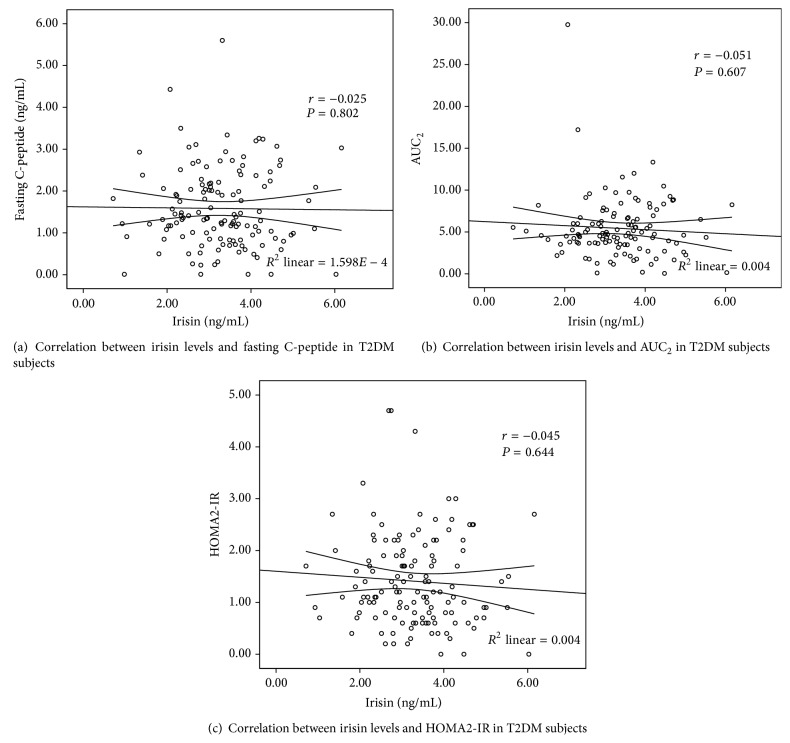
Correlation between irisin levels and glucose-related variables in T2DM subjects.

The correlations between irisin levels and glucose-related variables based on the values of 120 individual T2DM subjects were shown in Table 4 and Figure 2. Similar to the betatrophin, irisin levels were not associated with *β*-cell function either. However, it is positively associated with HOMA2-%S, even after controlling multiple covariates.

### 3.5. Correlations between Betatrophin and Irisin Levels in T2DM Subjects

**Figure 3:**
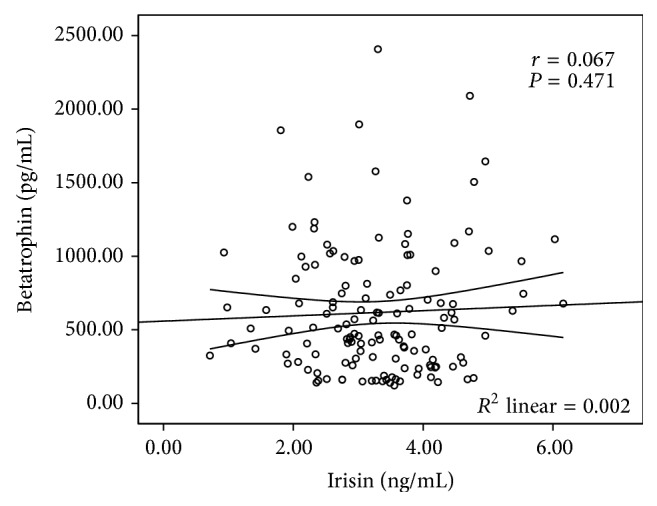
Correlation between irisin and betatrophin levels in T2DM subjects.

Finally, as shown in Figure 3, we analysed the correlation between betatrophin and irisin levels in T2DM subjects. Unfortunately, no significant relationship was observed between these two hormones.

## 4. Discussion

For lacking direct evidence of the role of betatrophin in islet *β*-cell replication in the human model and the fact that there were relatively few clinical studies focusing on the above issue, we first measured the circulating betatrophin levels in age-, sex- and BMI-matched healthy NGT and T2DM subjects. Similar to what has been previously reported [15], betatrophin levels were almost doubled in patients with T2DM compared with NGT (592.1 ± 37.5 versus 291.1 ± 37.3 pg/mL). It is noteworthy that the levels of betatrophin reported by Espes et al. [16] and Chen et al. [17] were higher than those of ours despite the similar trends between T2DM and control subjects. These discrepancies might be due to the different age of subjects. As what was suggested by our study and the results of other studies [15, 16, 18], age seemed to be positively related to the levels of betatrophin. Age of the diabetic subjects in the studies of Chen et al. (60.7 ± 0.9 years) and Espes et al. (61.9 ± 1.7 years) is older than those of ours (56.3 ± 1.2 years) and thus might partially explain the difference of the results.

Moreover, we found there was no significant correlation between betatrophin and indices of *β*-cell function (AUC_0–0.5_, AUC_0-1_, AUC_0–2_, and HOMA2-%B), Futhermore, no relationships between betatrophin and glycemic control indices such as FBG and HbA1c were found. Suggesting that betatrophin might not play an important role in regulating glucose homeostasis, which had been proved by mice models from Wang and colleagues [6]. They reported that mice knocked out for ANGPTL8/betatrophin showed no alterations in glucose homeostasis when fed either chow or high fat diet. Then more recently, Gusarova and colleagues further confirmed this issue by overexpressing betatrophin in mice livers and observed no significant alteration in *β*-cell expansion nor glucose metabolism [7]. Nevertheless, these results were obtained from diet or S961-induced insulin resistant mice models; the role of betatrophin on *β*-cell expansion under more extreme conditions of *β*-cell destruction is still under a veil, such as in diet or gene-deficient T2DM models. Furthermore, the results in mice cannot be fully applied to humans. Jiao et al. observed that betatrophin of mice failed to induce human *β*-cell replication, which raised a possibility that mouse and human betatrophin might undergo different posttranslational processing [19, 20]. Additionally, Chen et al. [17] claimed that circulating betatrophin levels were associated with markers of insulin resistance (HOMA-IR, quantitative insulin sensitivity check index (QUICKI), the Gutt insulin sensitivity index (ISI_G_) and the Matsuda insulin sensitivity index (ISI_M_)); however, correlation between betatrophin and HOMA2-IR or HOMA2-%S is not observed in our population. These inconformities might be due to the different population we chosen. Different from the newly diagnosed T2DM patients, our subjects had a mean diabetic history of 9 years and are under antidiabetic treatment. Medications such as metformin, TZD, and exogenous insulin could potentially alter the degree of insulin resistance and therefore affect the relevance between betatrophin and insulin resistance.

The ability of irisin to induce browning of WAT is of considerable interest for research on obesity, diabetes, and general metabolism [21–23]. Therefore, since its discovery, numerous studies have reported on the association of irisin with metabolic diseases in human cohorts. Recently, a meta-analysis revealed that significantly lower levels of circulating irisin were present in patients with T2DM, indicating the possible important role of irisin in regulating glucose homeostasis [24]. Similar phenomenon was also observed in this study as expected. However, the correlation between irisin and pancreatic *β*-cell function remains unclear. A recently published article found that serum irisin levels were closely related to HOMA-*β* in NGT subjects, but they did not observe a similar relationship in diabetes subjects. So researchers suggested that irisin might promote insulin secretion by increasing the proliferation of *β*-cells and the absence of such a correlation in T2DM patients might be due to the limited sample size (*n* = 60) [9]. However, by enlarging the sample size and applying the AUC of C-peptide to assess pancreatic *β*-cell function, which is the gold standard measure so far [11], our study still could not find a significant association between irisin levels and pancreatic *β*-cell function. So we speculated that irisin may correlate with pancreatic *β*-cell function in NGT subjects, but after a person has gotten diabetes, the circulating irisin levels will not have enough capacity to significantly affect pancreatic *β*-cell function in such a disorder internal environment. Besides, contradictory to the results of Zhang et al. [10], we found betatrophin and irisin levels lacked a significant correlation in T2DM subjects, which might remind us to be very careful when applying the results from animal models to humans.

Additionally, similar to what has been reported [25], we found that irisin is positively associated with insulin sensitivity (HOMA2-%S), even after controlling multiple covariates such as age, gender, BMI, blood lipids, antidiabetic medications, and the duration of diabetes. However the previous findings about the association between irisin and insulin resistance are controversial in different researches. Park et al. [23] reported that circulating irisin levels were associated positively with HOMA-IR. However, Al-Daghri et al. [26] conducted a study in a cohort of 153 Saudi Arabian children and found in girls, but not in boys, HOMA-IR correlated negatively with irisin levels. These above results suggested the relationship between irisin and insulin resistance need to be further warranted, especially in T2DM subjects.

All our data indicates that (1) betatrophin may not control pancreatic *β*-cell expansion or regulate pancreatic *β*-cell function in T2DM patients; (2) irisin may not promote pancreatic *β*-cell proliferation or regulate pancreatic *β*-cell function through promoting the expression of betatrophin in humans; (3) the beneficial effect of irisin on glucose homeostasis may be due to other mechanisms, such as inducing browning of WAT [8, 10], rather than the direct effect on *β*-cell function.

The strengths of our study were that we used AUC of C-peptide during 75-g OGTT to reflect pancreatic *β*-cell function, which is the gold standard measure of *β*-cell function [11]. Both the acute insulin response (AUC_0–0.5_ and AUC_0-1_) and the whole *β*-cell capacity (AUC_0–2_) were analysed in our study. Meanwhile, our study has some limitations. First, a cross-sectional study could not infer causality between these two hormones and *β*-cell function. Second, we included previously diagnosed T2DM patients; the medication history, especially insulin usage, may affect results. However, the indexes of *β*-cell function and insulin resistance were calculated based on C-peptide instead of insulin, which ensured the accuracy of results as far as possible. Third, as betatrophin and irisin levels are not just related to *β*-cells but also adipocytes and other cells, betatrophin and irisin levels may correlate with other adipokines or cytokines such as leptin [27, 28]. These factors were not adjusted in our model and thus might influence our results. Finally, HOMA2-%S and HOMA2-IR could not accurately reflect insulin sensitivity and insulin resistance. The relationships between these two hormones and insulin resistance need further investigation.

## 5. Conclusion

In conclusion, we have found that the circulating levels of betatrophin were significantly elevated, while irisin levels were significantly decreased, in patients with T2DM compared to NGT subjects. However, circulating betatrophin levels were not associated with *β*-cell function and insulin resistance in previously diagnosed T2DM patients, while irisin correlated positively with insulin sensitivity but is not associated with *β*-cell function-related variables. Considering the complexity of the mechanisms of betatrophin and irisin functioning, we cannot exclude the possibility that the betatrophin and irisin levels might be associated with other unknown intermediate factors, which may affect *β*-cell function and insulin resistance. Further studies, especially those with histology evidences, are needed to demonstrate the associations of betatrophin and irisin with *β*-cell function in diabetic subjects.

## Figures and Tables

**Table 1:** Characteristics of NGT and T2DM subjects.

Characteristics	NGT (*n* = 20)	T2DM (*n* = 120)	*P*-value
Female [*n* (%)]	10 (45.5%)	52 (43.3%)	0.750
Age (years)	54.4 ± 2.9	56.3 ± 1.2	0.232
BMI (kg/m^2^)	26.0 ± 0.5	26.3 ± 0.4	0.340
Systolic BP (mmHg)	128.3 ± 4.0	132.8 ± 1.5	0.186
Diastolic BP (mmHg)	72.1 ± 2.6	76.7 ± 1.1	0.109
FBG (mmol/L)	5.3 ± 0.1	7.8 ± 0.3	**<0.001**
HbA1c (%)	5.3 ± 0.1	8.6 ± 0.2	**<0.001**
Fasting insulin (mIU/L)	4.90 (3.2–7.6)	12.0 (7.5–17.8)	**<0.001**
Fasting C-peptide (ng/mL)	1.5 ± 0.1	1.6 ± 0.1	0.424
Betatrophin (pg/mL)	291.1 ± 37.3	592.1 ± 37.5	**<0.001**
Irisin (ng/mL)	4.7 ± 0.1	3.4 ± 0.1	**<0.001**

The data are expressed as the means ± SEM or median (interquartile range) or numbers (%). NGT, normal glucose tolerance; T2DM, type 2 diabetes mellitus; BMI, body mass index; BP, blood pressure; FBG, fasting blood glucose.

**Table 2:** Characteristics of the study participants by AUC of C-peptide quartiles in T2DM subjects.

Characteristics	Quartile 1 (*n* = 30)	Quartile 2 (*n* = 30)	Quartile 3 (*n* = 30)	Quartile 4 (*n* = 30)	*P*-value^a^
Female [*n* (%)]	18 (60.0%)	12 (40.0%)	9 (30%)	13 (43.3%)	0.127
Age (years)	61.6 ± 2.0	54.0 ± 2.6^b^	53.8 ± 2.4^b^	55.8 ± 3.0^b^	0.098
BMI (kg/m^2^)	24.1 ± 0.8	25.6 ± 0.6	27.3 ± 0.8^b^	28.2 ± 0.9^bc^	**0.001**
Systolic BP (mmHg)	137.3 ± 3.3	129.1 ± 2.5	133.6 ± 3.7	131.4 ± 3.0	0.315
Diastolic BP (mmHg)	74.4 ± 2.3	74.5 ± 1.9	79.4 ± 2.9	78.6 ± 2.4	0.302
FBG (mmol/L)	7.8 ± 0.5	8.1 ± 0.5	7.7 ± 0.6	7.6 ± 0.4	0.527
HbA1c (%)	9.2 ± 0.4	8.9 ± 0.4	8.3 ± 0.3	8.1 ± 0.4^d^	**0.025**
Fasting insulin (mIU/L)	10.0 (5.1–15.4)	11.2 (6.3–16.8)	12.3 (8.6–16.5)	15.8 (11.1–20.1)	0.051
Fasting C-peptide (ng/mL)	0.6 ± 0.1	1.3 ± 0.1^b^	1.7 ± 0.1^bc^	2.6 ± 0.1^bcd^	**<0.001**
HOMA2-%B	21.7 (14.5–27.3)	39.7 (25.4–60.2)^b^	37.9 (29.1–63.0)^b^	82.9 (46.5–102.1)^bcd^	**< 0.001**
HOMA2-%S	166.7 (130.2–274.8)	96.7 (71.0–131.7)^b^	72.5 (51.9–96.2)^bc^	44.2 (38.6–59.0)^bcd^	**<0.001**
HOMA2-IR	0.6 (0.3–0.7)	1.0 (0.8–1.4)^b^	1.4 (1.1–1.9)^bc^	2.3 (1.7–2.6)^bcd^	**<0.001**
Duration of diabetes (years)	13.2 ± 1.6	7.0 ± 1.1^b^	7.8 ± 1.4^b^	6.0 ± 1.0^b^	**0.001**
Insulin secretagogues treatment [*n* (%)]	11 (36.7%)	17 (56.7%)	20 (66.7%)	17 (56.7%)	0.110
Other antidiabetic medications [*n* (%)]	23 (76.7%)	21 (70.0%)	28 (93.3%)	26 (86.7%)	0.096
Insulin treatment [*n* (%)]	23 (76.7%)	16 (53.3%)	9 (30.0%)^b^	5 (16.7%)^bc^	**<0.001**
Triglyceride (mmol/L)	1.1 (0.7–1.5)	1.6 (1.1–2.3)^b^	1.7 (1.0–2.1)^b^	1.9 (1.4–2.6)^b^	**<0.001**
Cholesterol (mmol/L)	4.9 ± 0.2	4.6 ± 0.2	4.5 ± 0.2	5.0 ± 0.2	0.191
HDL-C (mmol/L)	1.5 ± 0.1	1.2 ± 0.1^b^	1.3 ± 0.1^b^	1.2 ± 0.0^b^	**0.001**
LDL-C (mmol/L)	2.8 ± 0.2	2.7 ± 0.1	2.6 ± 0.1	3.0 ± 0.1	0.217
Betatrophin (pg/mL)	637.0 ± 92.3	522.2 ± 70.1	633.9 ± 88.2	575.6 ± 70.4	0.720
Irisin (ng/mL)	3.6 ± 0.2	3.1 ± 0.2	3.1 ± 0.2	3.6 ± 0.2	0.073

The data are expressed as the means ± SEM or median (interquartile range) or numbers (%). AUC, area under the curve; BMI, body mass index; BP, blood pressure; FBG, fasting blood glucose; HOMA2-%B, homoeostasis model assessment of *β*-cell function; HOMA2-%S, homoeostasis model assessment of insulin sensitivity; HOMA2-IR, homoeostasis model assessment of insulin resistance; HDL-C, high-density lipoprotein cholesterol; LDL-C, low-density lipoprotein cholesterol. ^a^Difference between four groups; ^b^ *P* < 0.05 compared with Quartile 1 group; ^c^ *P* < 0.05 compared with Quartile 2 group; ^d^ *P* < 0.05 compared with Quartile 3 group.

**Table 3:** Partial correlations between betatrophin levels and glucose-related variables in T2DM subjects.

Characteristics	Model 1		Model 2		Model 3	
	*r*	*P*-value	Partial *r *	*P*-value	Partial *r *	*P*-value
HbA1c	0.026	0.765	0.129	0.174	0.057	0.566
FBG	−0.112	0.194	−0.053	0.577	−0.044	0.659
Fasting insulin	−0.080	0.354	−0.044	0.644	−0.103	0.296
Fasting C-peptide	0.019	0.828	0.015	0.878	0.035	0.719
AUC_0.5_	0.007	0.936	0.032	0.738	0.058	0.555
AUC_1_	0.005	0.961	0.023	0.809	0.056	0.572
AUC_2_	−0.007	0.940	−0.002	0.984	0.035	0.726
HOMA2-%B	0.130	0.140	0.087	0.363	0.080	0.419
HOMA2-%S	0.028	0.753	0.044	0.642	0.037	0.709
HOMA2-IR	−0.015	0.865	0.005	0.954	0.016	0.872

Model 1: unadjusted.

Model 2: adjusted for age, gender, and BMI.

Model 3: adjusted for age, gender, BMI, triglyceride, cholesterol, use of insulin, insulin secretagogues treatment, other antidiabetic medications, and duration of diabetes.

**Table 4:** Partial correlations between irisin levels and glucose-related variables in T2DM subjects.

Characteristics	Model 1		Model 2		Model 3	
	*r *	*P*-value	Partial *r *	*P*-value	Partial *r *	*P*-value
HbA1c	0.062	0.481	0.083	0.383	0.080	0.415
FBG	−0.166	0.053	−0.044	0.646	−0.069	0.486
Fasting insulin	0.031	0.720	0.012	0.899	0.031	0.751
Fasting C-peptide	−0.013	0.886	−0.025	0.796	−0.025	0.803
AUC_0.5_	−0.091	0.318	−0.071	0.455	−0.067	0.497
AUC_1_	−0.085	0.349	−0.069	0.470	−0.066	0.505
AUC_2_	−0.065	0.481	−0.047	0.620	−0.051	0.607
HOMA2-%B	−0.050	0.569	−0.061	0.520	−0.021	0.828
HOMA2-%S	**0.243**	**0.005**	**0.225**	**0.017**	**0.218**	**0.026**
HOMA2-IR	−0.066	0.454	−0.031	0.745	−0.045	0.647

Model 1: unadjusted.

Model 2: adjusted for age, gender, and BMI.

Model 3: adjusted for age, gender, BMI, triglyceride, cholesterol, use of insulin, insulin secretagogues treatment, other antidiabetic medications, and duration of diabetes.
